# New Appliances and Things Medical

**Published:** 1898-11-26

**Authors:** 


### NEW APPLIANCES AND THINGS MEDICAL.

[We shall be glad to receive, at our Office, 28 & 29, Southampton Street, Strand, London, W.C., from the manufacturers, specimens of all new preparations and appliances which may be brought out from time to time.]

### SOL. IODI. (DOWNES.)

(Brooks and Co., 136, Lower Baggot Street, Dublin.)

This is a solution of iodine which, although capable of being quickly absorbed into the system, as shown by its being excreted, does not produce that irritation and induration of the skin which follows the application of most iodine preparations. In fact, it hardly discolours the skin. Thus it is likely to be of much service where it is desired that iodine should be locally absorbed, as, for example, in various glandular affections, while on the other hand, where stimulating and counter-irritant effects are desired, the ordinary preparations, such as the liniment of iodine, are to be preferred.

### OXYDOL (EAU MAICHE).

(Maiche, Limited, St. Mary Axe, E.C.)

Oxydol is stated to be "chemically pure water containing three times its volume of available oxygen and to be absolutely harmless." Moreover, when oxydol comes in contact with the constituents of the blood or with the internal mucous membrane oxygen is slowly given off. Oxydol is an inodorous and colourless aqueous solution containing one of the most active of known oxygen-yielding bodies, which has been found efficacious as a dressing for purulent discharges on account of its powerful antiseptic properties. Moreover, it is painless, does not stain, and is non-poisonous. It has also been suggested for use in diabetes, gout, and rheumatism.

